# Long-term follow-up of pancreatic islet transplantation in a patient with Wolfram syndrome: a case report

**DOI:** 10.1007/s00592-025-02558-4

**Published:** 2025-07-30

**Authors:** Davide Catarinella, Costanza Festorazzi, Rossana Caldara, Lorenzo Piemonti

**Affiliations:** 1 Diabetes Research Institute and Clinic Unit of Regenerative Medicine and Organ Transplants, IRCCS Ospedale San Raffaele, Milan, Italy; 2 Università Vita-Salute San Raffaele, Milan, Italy; 3 Diabetes Research Institute, IRCCS Ospedale San Raffaele, Via Olgettina 60, 20132 Milan, Italy

**Keywords:** Beta-cell replacement, Glycemic control, Islet transplantation, Monogenic diabetes, Neurodegeneration, Pancreatic Islets, Stem cell therapy, Tacrolimus, Wolfram syndrome

## Introduction

Wolfram syndrome is a progressive monogenic disorder resulting from mutations in the WFS1 gene, which encodes wolframin, a protein essential for endoplasmic reticulum function [1]. The condition presents a wide spectrum of symptoms, including diabetes mellitus, optic atrophy, diabetes insipidus, and neurodegeneration Insulin-dependent diabetes is often the earliest clinical manifestation, typically appearing within the first decade of life, with onset ranging from infancy to 17 years [2]. Unlike type 1 autoimmune diabetes mellitus, the diabetes in Wolfram syndrome stems from a primary beta-cell defect rather than autoimmune destruction. This creates unique management challenges, as conventional therapies do not address the underlying cellular dysfunction or the progressive loss of beta cells. Although the use of GLP-1 receptor agonists is under investigation [3], nearly all affected individuals require insulin therapy. While diabetes in Wolfram syndrome is generally mild and associated with a lower risk of microvascular complications compared to autoimmune diabetes, some patients may experience” brittle” diabetes, marked by severe glycemic fluctuations and frequent episodes of hypoglycemia and hyperglycemia [4].

Isolated pancreatic islet transplantation has proven effective in achieving optimal glycemic control and, in many cases, insulin independence in patients with autoimmune type 1 diabetes, particularly those characterized by severe glycemic fluctuations and frequent episodes of hypoglycemia and hyperglycemia [5]. Traditionally focused on autoimmune diabetes, beta cell replacement has recently garnered interest as a therapeutic option for broader forms of insulin-deficient diabetes, including non-autoimmune and monogenic disorders. Advances in cellular therapies, such as islets derived from pluripotent stem cells, have further expanded the potential applications of this approach. Theoretically, there is no reason an islet transplantation approach should not be considered in the presence of autoantibody-negative insulinopenic diabetes, even when the underlying cause is not autoimmune. On the contrary, the absence of autoimmunity is a potential advantage, as it could improve the survival of the transplanted islets. This report highlights the case of an adult with Wolfram syndrome who underwent islet transplantation, proving its feasibility and potential benefits in managing monogenic diabetes, where the lack of autoimmunity could contribute to superior outcomes.

### Case presentation

**Fig. 1:**
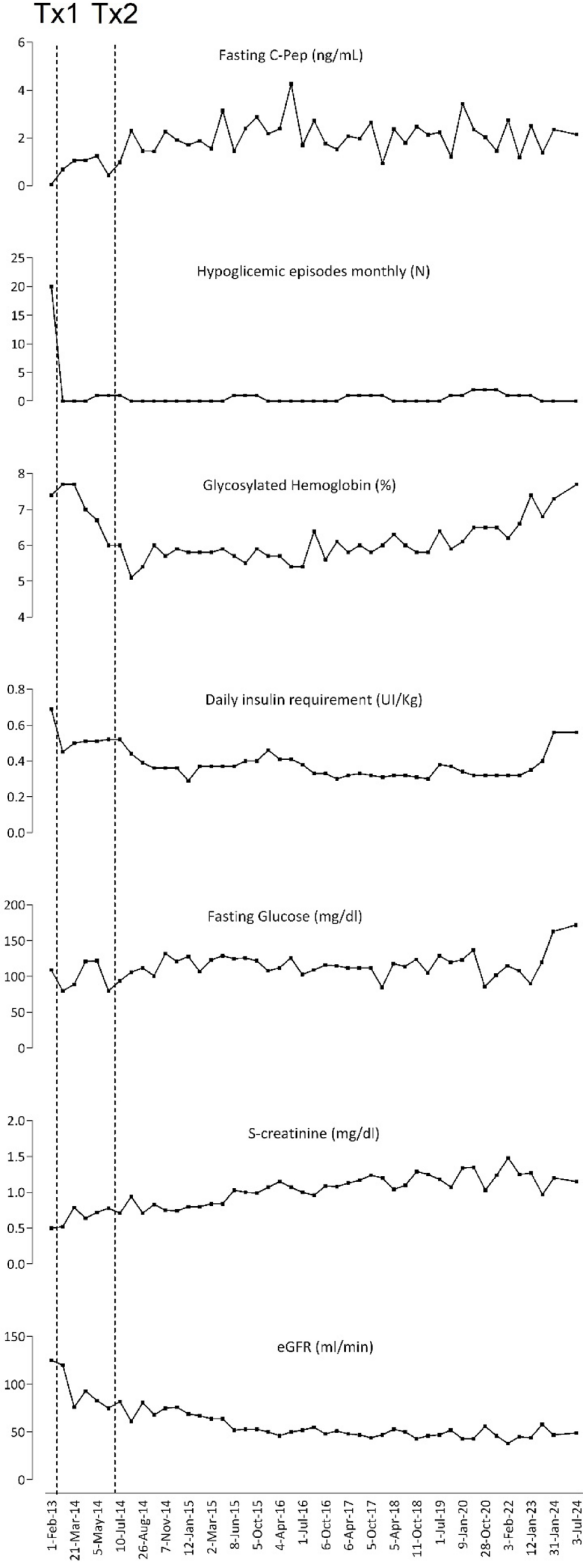
Dynamic changes in metabolic control and renal function before and after the first (Tx1) and second (Tx2) pancreatic islet transplantations. From top to bottom: fasting C-peptide levels (ng/mL); monthly frequency of hypoglycemic episodes (number); insulin requirement adjusted for body weight (IU/kg); glycosylated hemoglobin (HbA1c in %); fasting glucose levels (mg/dL); serum creatinine (mg/dL); estimated glomerular filtration rate (eGFR in mL/min). The x-axis represents time points from February 2013 to July 2024, showing the measurement dates

A 59-year-old woman with a reported diagnosis of type 1 diabetes presented in 2012 to the Department of Transplantation at San Raffaele Hospital for evaluation of potential pancreatic islet transplantation. The patient had a long-standing history of type 1 diabetes, diagnosed in 1976 at the age of 17, and was admitted for evaluation of eligibility for pancreatic islet transplantation in 2012 due to unstable glycemic control and diabetes-related complications. In 2005, she transitioned from a basal-bolus insulin regimen to continuous subcutaneous insulin infusion (CSII) via insulin pump, which initially led to significant improvement in glycemic control. In the year preceding transplantation, her glycemic control had worsened, with marked intraday glucose fluctuations (ranging from 50 to 400 mg/dL), and an HbA1c of 7.6%. She reported frequent symptomatic hypoglycemic episodes (3–4 per month), typically perceived when glucose levels dropped below 50 mg/dL. Her insulin therapy at the time consisted of pump-based delivery with a basal dose of 18 IU/day and boluses of 2, 6, and 6 IU at meals. Although no diabetic nephropathy was documented, she had been on low-dose ACE inhibitor therapy for nephroprotection for several years. This clinical profile — characterized by labile glycemia, risk of hypoglycemia, and multiple chronic complications (including optic nerve atrophy, neurogenic bladder, and peripheral neuropathy) — strongly supported the indication for islet transplantation. Laboratory tests revealed the absence of anti-GAD, anti-IA2, anti-insulin, and anti-ZNT8 antibodies, while fasting and stimulated C-peptide levels could not be measured. Her weight at the time of evaluation was 62 kg. On March 8, 2014, the patient underwent her first pancreatic islet transplantation (IEQ 346725; IEQ/kg 5592). Induction therapy included basiliximab (20 mg at day 0 and 4) alongside reparixin (an anti-CXCR1/2 agent) 2.772 mg/kg body weight/h, initiated on day 0 and continued for 7 days. Maintenance immunosuppression consisted of tacrolimus (target trough level: 3–6 ng/mL) and sirolimus (target trough level: 12–15 ng/mL for the first 1–3 months; 10–12 ng/mL thereafter). Four months later, on July 3, 2014, a second islet infusion was performed (IEQ 246967; IEQ/kg 3983), with the same post-transplantation therapy regimen as the first infusion. However, the procedure was complicated by the development of hemoperitoneum, which needed urgent laparoscopic intervention, successfully resolving the complication. A 10-year follow-up is available, during which significant improvements in glycemic control and sustained islet function were seen. Over time, both fasting and stimulated C-peptide levels gradually increased, enabling the patient to significantly reduce her daily insulin requirement, leading to a substantial improvement in glycemic control. Additionally, the frequency of hypoglycemic episodes decreased markedly, with only one non-severe episode reported per month (Figs. 1).

**Fig. 2:**
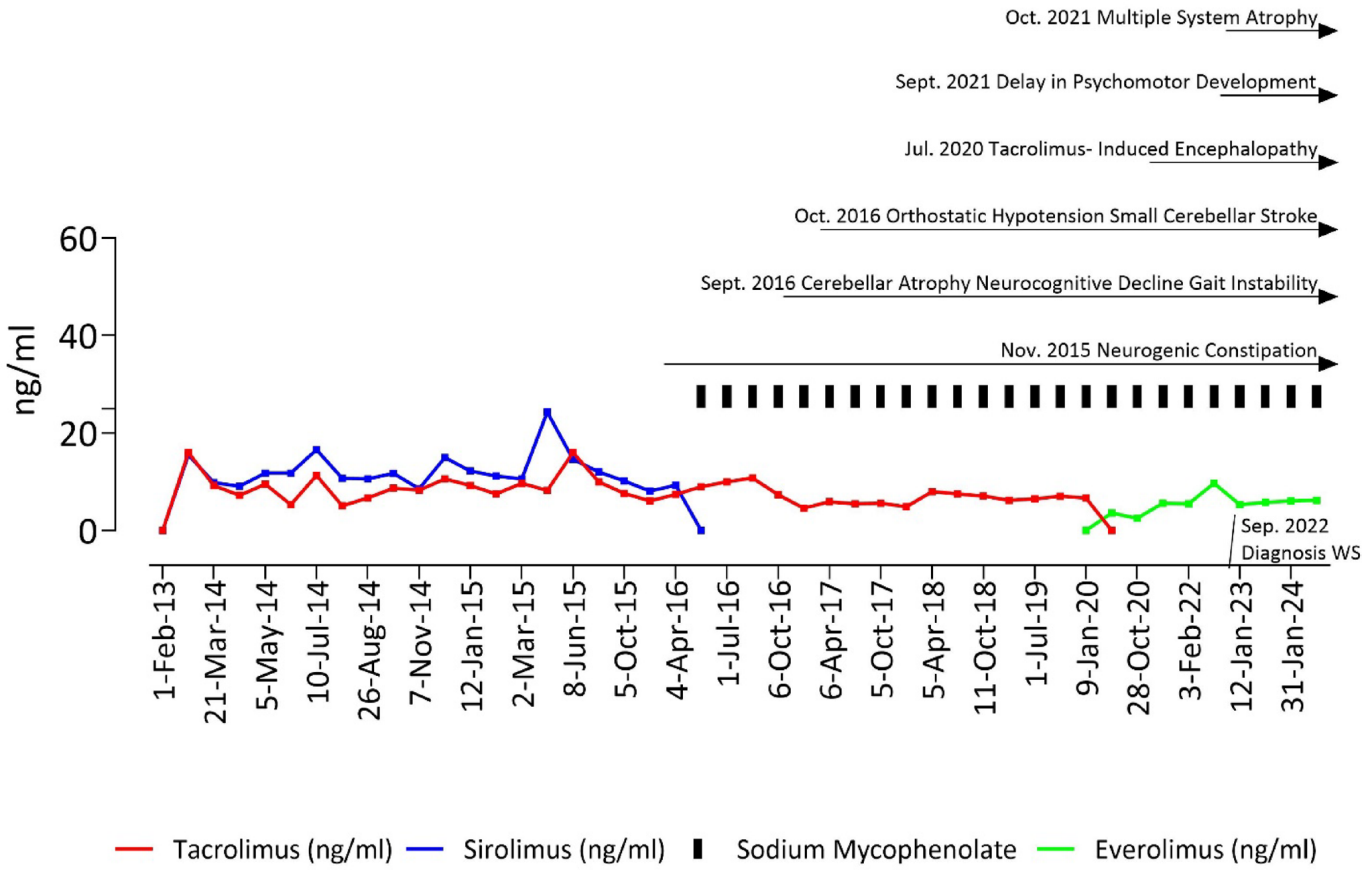
Changes in drug regimens and monitoring of therapeutic levels during the study period, alongside the progression of Wolfram Syndrome symptoms. Time points on the x-axis span from February 2013 to January 2024. The concentration levels (ng/mL) of three immunosuppressive agents are shown over time: tacrolimus (red line), sirolimus (blue line), sodium mycophenolate (fixed dosage of 360 mg twice daily, black columns), and everolimus (green line). The timeline also highlights the progression of symptoms associated with Wolfram Syndrome

Despite the positive metabolic outcomes, the patient’s neurological and cognitive functions declined. (Fig. 2). In early 2016, she began experiencing recurrent episodes of orthostatic hypotension, asthenia, and arthralgia. Therefore, sirolimus was dropped in favor of mycophenolate sodium (360 mg twice daily) with partial beneficial effect. A brain MRI revealed a chronic ischemic area in the left cerebellar region, and autonomic testing showed cardiovascular autonomic dysfunction, leading to a diagnosis of diabetic autonomic neuropathy. In 2019, her balance and gait deteriorated further, resulting in hospitalization at the Transplant Medicine department of San Raffaele Hospital. Brain MRI showed an atrophic pattern in the pontocerebellar region and posterior supratentorial leukoencephalopathy, likely due to chronic vasculopathy. Given her ongoing immunosuppressive therapy following earlier islet transplantations, tacrolimus-induced encephalopathy was suspected. Tacrolimus was stopped and replaced with everolimus (target trough level 3–8ng/mL), but this change did not alleviate the neurological symptoms (Fig. 2). A comprehensive neurological evaluation revealed impairments in visuospatial abilities and cognitive function. Electromyography showed mild chronic axonal sensory-motor polyneuropathy, primarily affecting the distal lower limbs. Autonomic testing confirmed dysfunction in the autonomic nervous system, especially in the cardiovascular domain, consistent with prior findings of orthostatic hypotension. Neuropsychological assessments showed that cognitive function remained largely within normal limits (MMSE 26/30), though deficits were noted in anterograde memory (both verbal and visual), attentional-executive function, and logical reasoning. Additionally, verbal comprehension and graphic planning abilities were impaired. A review of all available records, both before and after the transplantation, showed consistent negativity for anti-GAD, IA-2, ZnT8, and insulin antibodies. The clinical presentation raised suspicion of Wolfram syndrome, prompting genetic testing. The patient was found to carry the c.1839G > A, p.Trp613* (ENST00000503569.1) variant in the WFS1 gene in a heterozygous state. Based on these findings, the patient was reclassified as having classical Wolfram syndrome.

## Discussion

This case involves a 59-year-old woman with a longstanding history of diabetes who underwent pancreatic islet transplantation and was later diagnosed with Wolfram syndrome. The transplantation led to notable metabolic improvements, including better glycemic control, reduced insulin requirements, and a decrease in severe hypoglycemic episodes. These outcomes provide long-term evidence of effective metabolic management in an insulinopenic patient without autoimmune diabetes, highlighting that islet transplantation can be successful even in the absence of an autoimmune cause for diabetes.

The diagnosis of Wolfram syndrome was made only after neurological symptoms appeared, highlighting the challenge of recognizing it in patients with diabetes-related complications. Her clinical course, characterized by chronic diabetes, recurrent hypoglycemia, autonomic dysfunction, and progressive neurological issues, underscores the complexity of managing diabetes in the context of rare genetic disorders. The diagnosis of her diabetes became increasingly difficult with disease progression, and the neurological decline added further complexity. The impact of improved glycemic control or immunosuppressive therapy on neurological symptoms is still unclear, emphasizing the challenges of managing such multifaceted conditions.

A previous report described pancreatic transplantation in a patient with Wolfram syndrome, but with only six months of follow-up [4]. In contrast, this case provides the first long-term follow-up evidence of both graft survival and significant metabolic improvement in a patient with insulinopenic, non-autoimmune diabetes. This suggests that beta-cell replacement therapy could be a viable treatment option for such conditions, especially with the potential future use of pluripotent stem cells, including autologous ones, for islet replacement therapy.

In conclusion, this case proves that islet transplantation can offer long-term metabolic benefits for insulinopenic patients, even without an autoimmune origin. It also emphasizes the diagnostic and management challenges in evolving genetic conditions like Wolfram syndrome and highlights the potential of beta-cell replacement therapies, especially with advances in stem cell technology.

## Data Availability

Individual participant data will not be made available. Study protocol, statistical analysis plan, and analytical code will be available from the time of publication in response to any reasonable request addressed to the corresponding author.
